# KIF1A-Associated Neurological Disorder: An Overview of a Rare Mutational Disease

**DOI:** 10.3390/ph16020147

**Published:** 2023-01-19

**Authors:** Ayushi Nair, Alosh Greeny, Rajalakshmi Rajendran, Mohamed A. Abdelgawad, Mohammed M. Ghoneim, Roshni Pushpa Raghavan, Sachithra Thazhathuveedu Sudevan, Bijo Mathew, Hoon Kim

**Affiliations:** 1 Department of Pharmacy Practice, Amrita School of Pharmacy, Amrita Vishwa Vidyapeetham, Amrita Health Science Campus, Kochi 682041, India; 2 Department of Pharmaceutical Chemistry, College of Pharmacy, Jouf University, Sakaka, Al Jouf 72341, Saudi Arabia; 3 Department of Pharmaceutical Organic Chemistry, Faculty of Pharmacy, Beni-Suef University, Beni-Suef 62514, Egypt; 4 Department of Pharmacy Practice, College of Pharmacy, AlMaarefa University, Ad Diriyah 13713, Saudi Arabia; 5 Department of Pharmaceutical Chemistry, Amrita School of Pharmacy, Amrita Vishwa Vidyapeetham, AIMS Health Sciences Campus, Kochi 682 041, India; 6 Department of Pharmacy, and Research Institute of Life Pharmaceutical Sciences, Sunchon National University, Suncheon 57922, Republic of Korea

**Keywords:** KAND, *KIF1A* gene, microtubule, kinesin motor protein, neurological disorder

## Abstract

KIF1A-associated neurological diseases (KANDs) are a group of inherited conditions caused by changes in the microtubule (MT) motor protein KIF1A as a result of *KIF1A* gene mutations. Anterograde transport of membrane organelles is facilitated by the kinesin family protein encoded by the MT-based motor gene *KIF1A*. Variations in the *KIF1A* gene, which primarily affect the motor domain, disrupt its ability to transport synaptic vesicles containing synaptophysin and synaptotagmin leading to various neurological pathologies such as hereditary sensory neuropathy, autosomal dominant and recessive forms of spastic paraplegia, and different neurological conditions. These mutations are frequently misdiagnosed because they result from spontaneous, non-inherited genomic alterations. Whole-exome sequencing (WES), a cutting-edge method, assists neurologists in diagnosing the illness and in planning and choosing the best course of action. These conditions are simple to be identified in pediatric and have a life expectancy of 5–7 years. There is presently no permanent treatment for these illnesses, and researchers have not yet discovered a medicine to treat them. Scientists have more hope in gene therapy since it can be used to cure diseases brought on by mutations. In this review article, we discussed some of the experimental gene therapy methods, including gene replacement, gene knockdown, symptomatic gene therapy, and cell suicide gene therapy. It also covered its clinical symptoms, pathogenesis, current diagnostics, therapy, and research advances currently occurring in the field of KAND-related disorders. This review also explained the impact that gene therapy can be designed in this direction and afford the remarkable benefits to the patients and society.

## 1. Introduction

KIF1A-associated neurological diseases (KAND) are a group of neurological illnesses caused by changes in the microtubule (MT) motor protein KIF1A as a consequence of a *KIF1A* gene mutation. These genetic changes might produce pathogenic mutations and lead to neurological disorders in patient [1]. Due to the limited availability of full gene sequencing and exome sequencing at the time, these illnesses were originally discovered in 2011, and many patients received the wrong diagnosis [2]. There are various KAND variations that can be passed down both dominantly and recessively. Researchers have noted various types of mutations in the same proteins in patients, and intriguingly, the majority of them exhibit private variants, opening up the possibility for further investigation and the discovery of further variants in these KIF1A-related disorders (KRD) [3]. This uncommon illness, which can also damage the vision, muscles, and nerves, primarily targets the neurons in the brain [4]. Studies on metabolic diseases, such as diabetes mellitus, have found that the *KIF1A* gene is more expressed and more immunoreactive [5]. Furthermore, this might eventually cause other neurological issues including encephalopathy and brain shrinkage [6].

**Figure 1:**
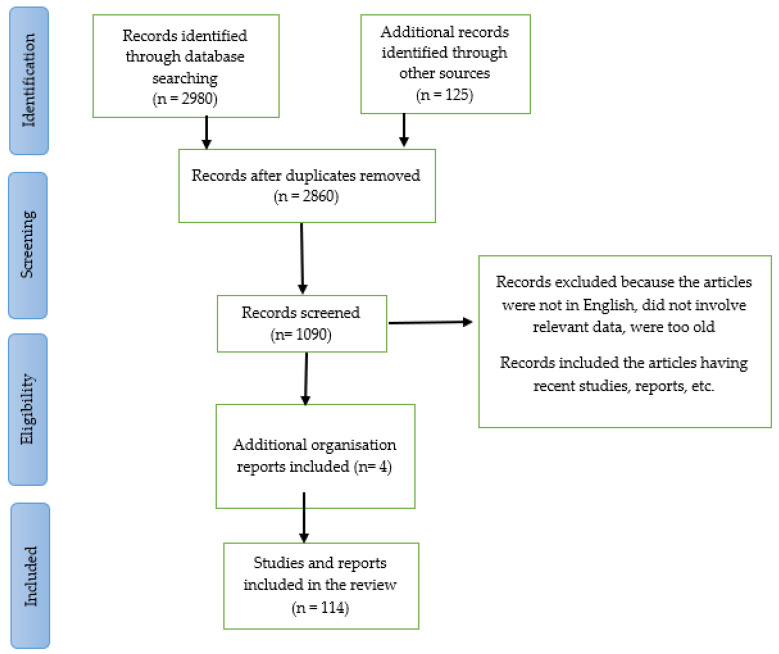
Work flow of this review and systematic review.

The molecular motor KIF1A affects the survival and development of sensory neurons in our body as well as the movement of membrane-bound cargo [7]. If there are any disturbances in these neuronal trafficking pathways, which are strictly controlled due to the functional compartmentalization of neurons and connect the neuron’s body, dendrites, and axons, neurodegenerative diseases would result [8]. The KIF1A protein’s normal function can be altered by one mutation or numerous mutations in the gene that codes for it, resulting in this disorder [3]. Due to the serious life-threatening problems brought on by genetic abnormalities in this condition, patients’ quality of life and life expectancy might be greatly affected [2]. The detailed work flow of current work is outlined in PRISMA diagram (Figure 1)

## 2. KIF1A Gene

Kinesin-like proteins, better known as axonal transporter of the synaptic vesicles, are MT-centered motors belonging to the kinesin family of proteins, and are involved in anterograde transport of some major membrane organelles, vesicles, macro-protein, and mRNA along the microtubular structures. This in itself explains that the *KIF1A* gene plays an important role in axonal transport as well as meiosis and mitosis processes [8]. The KIF1A is also required for neuronal dense core vesicles (DCV) transport to the dendritic spines and axons [9].

**Figure 2:**
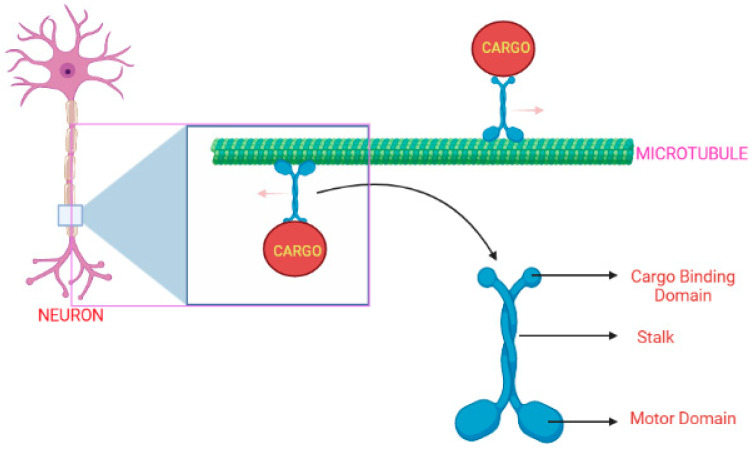
The KIF1A protein’s typical shape and movement along the MT surface in a healthy neuronal cell [15]. (Created with Biorender.com).

KIF1A protein comprises a neck region, a tail, and an N-terminal motor domain, as illustrated in Figure 2 [10]. The motor-domain has MT-dependent ATPase activity and MT-binding actions, whereas the tail consists of a stalk domain for protein binding, and a pleckstrin-homology [PH] domain is used for lipid binding. A small strand “neck linker” and the neck coil regions play an important role in the dimerization process of kinesin-3-motor and the processive motility [11,12,13]. The *KIF1A* gene initially exists as an inactive dimer and this stage is maintained by autoinhibitory mechanisms. It gets activated when bound to cargo, forming a homo-dimer enabling the transport of synaptic vesicle precursors along the MT [10,11,14].

**Figure 3:**
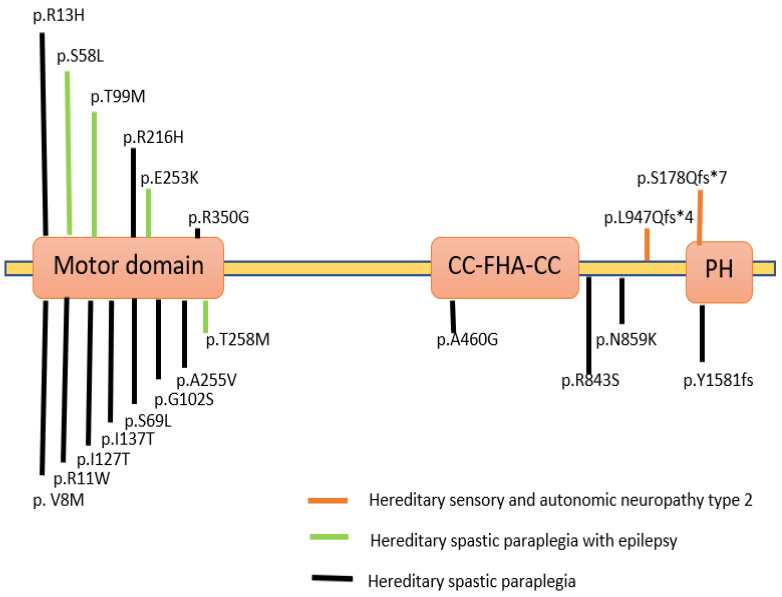
KIF1A protein depicted schematically, along with the locations of mutations in human KIF1A linked to several neurological diseases. The KIF1A subunit protein peptide contains these mutations in a variety of regions and domains [20,21,22,23]. (Created with Biorender.com).

Variations in the *KIF1A* gene have been associated with various clinical conditions and are described in a database known as Online Mendelian Inheritance in Man (OMIM). Variants in KIF1A were studied in various neurodegenerative diseases with dominant and recessive inheritance (Figure 3). In patients suffering from severe neurodegenerative disorders, homozygous recessive mutations in the *KIF1A* gene were first described as hereditary sensory and autonomic neuropathy type 2 and as three consanguineous families with an autosomal recessive form of hereditary spastic paraplegia (HSP) with an autosomal dominant form of SPG30 [16]. A particular mutation p.T99M was reported in patients having an intellectual disability (ID), spasticity and axial hypotonia [17]. A partially overlapping phenotype-brain atrophy with progressive encephalopathy was recently found to be associated with *de-novo* KIF1A mutations [6]. Several *de-novo* variations and mutations in patients were classified as either pure or complicated. The complicated type is accompanied by axonal neuropathy and brain cerebellar atrophy [18,19]. 

## 3. KIF1A—As a Super Engaging Motor

KIF1A protein has the unique ability to be kinetically tuned to become a super engaging motor that ensures its proper functioning and integrity under hindering conditions when under loads [24]. The ability of these kinesin motor proteins under mechanical loads is very important for the proper intracellular transport of the cargo. The high loads on the kinesin motor will affect the motor speed as well as the MT attachment lifetime. As the KIF1A belongs to the kinesin-3 family, the super processive behavior under zero loads of these proteins will purely depend upon the loops and cores of this proteins [10,25,26]. Serapion et al. and Allison et al. found that the processive runs done by the KIF1A get terminated when under load and thus low average termination forces are required as compared to KIF5B, by comparing two different motors, i.e., KIF1A and KIF5B. Hence, therefore, it shows that KIF1A uses a different mechanism to work under loads to increase its efficiency and is not similar to other kinesin motors. 

The distinct feature of KIF1A is its ability to form reengagement structures with the aid of different loops. Loop 12 plays a significant role in the motility of these proteins, and especially the positively charged K loop insert present in loop 12 has a crucial role [27,28,29,30]. In some studies, the scientists tried to replace the lysine (K) and remove the charge from loop 12 from the motor and this resulted in a lack of ability of the protein to work under load [24]. MT nucleotide and loop 12 influence the motor’s ability to reengage under mechanical load. The degree of expansion of the MT lattice and the polymerization of the MT with different nucleotides affects the rate at which the reengagement takes place [31,32,33]. Thus, all these studies reveal that the proper functioning of loop 12 and the arrangement of MT nucleotides aids in the adaptive nature of KIF1A and its novel mechanism of transport of cargo by super engagement and reengagement methods.

## 4. Symptoms

**Figure 4:**
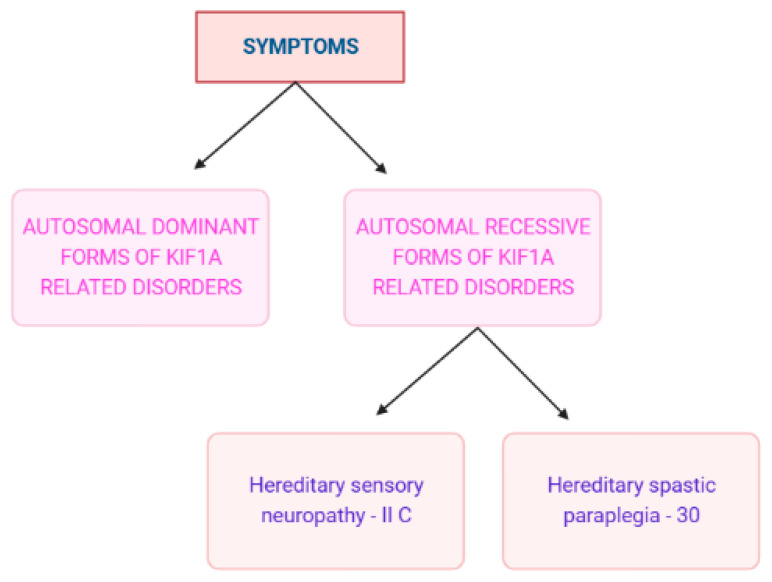
KAND symptoms on a clinical basis [36]. II C is a type of autosomal recessive disorder and can be represented as 2C also. (Created with BioRender.com.).

KAND has a broad phenotypic spectrum of signs and symptoms. Intellectual disability, spasticity, inherited progressive spastic paraplegia, cerebral atrophy, optic nerve atrophy, and microcephaly are a few of these (Figure 4). But most frequently, most individuals show signs of seizures [3]. Cerebellar function impairment has also been reported in some clinical investigations, and some patients have also shown dysautonomic symptoms such as temperature instability and urine retention. Due to gastrointestinal dysfunction, people with severe diseases could need parenteral nourishment [2]. Several studies state that the mutation in the KIF1A gene directly affects the motility of hetero-dimeric motors [3,34,35].

The following examples are the typical services that a KAND patient may require [36];

- Neurologist—neurological abnormality, seizures, and spasticity
- Ophthalmologist—impaired vision
- Pediatrician—developmental delay
- Specialized therapist—issues with speech and coordination
- A team of specialists—intellectual disability

### 4.1. Autosomal Dominant Variety of KRD [KIF1A-Related Disorders]

Developmental delays, cerebellar atrophy, peripheral neuropathy, ptosis, facial diplegia, intention tremors, strabismus, nystagmus, clumsiness, and ataxia are some of the typical symptoms. Other symptoms include hypertonia (increased muscle tone), hyperreflexia (exaggerated reflexes), spasticity (muscle tightness), and hyperreflexia [36,37]. 

### 4.2. Autosomal Recessive Forms of KIF1A-Related Disorders

#### 4.2.1. Hereditary Sensory Neuropathy IIC (Also Represented as 2C)

The deterioration of the neurons that results in the loss of feeling is the cause of this neuropathy. Other signs, such as numbness and tingling, are also detected and eventually lead to the loss of sensation. As a result of this affecting sensory neurons, automatic or involuntary body movements are also directly impacted [36,38,39]. 

#### 4.2.2. HSP

There are more than 80 genetically different types of HSP [40]. These types of spastic paraplegias are caused due to variations in the *KIF1A* gene and are referred to as an autosomal dominant type of Spastic Paraplegia (SPG30). The major symptoms include neurological difficulties, severe leg weakness, and spasticity [38,41,42]. The major sites of mutations in the human KAND protein are depicted in Figure 3.

## 5. Diagnosis

Traditional diagnostic methods such as multiplex probe amplification, karyotyping, genetic testing, and chromosomal microarray analysis were employed to screen out all forms of neurological disorders, including KAND [43,44,45,46,47]. The main drawback of these procedures was that different mutation types began exhibiting comparable clinical traits. A cutting-edge method called whole-exome sequencing (WES) is now becoming more widely used [48]. The WES aids in the diagnosis of the condition and the selection of the most effective treatment plan for the neurologist, particularly the pediatric neurologist [49]. Although WES is frequently used to diagnose KAND, it has several technical limitations that make it difficult to detect trinucleotide repeats, big indels, and epigenetic modifications that could impede the diagnosis of the illness [50,51]. Some of the newer fields such as the cytogenetics, chromosomal aberration, molecular diagnostic technique, carrier detection techniques are recently being explored by scientists to develop a novel method for the diagnosis of KAND.

## 6. Treatment

There is currently no effective therapy or cure for KAND. However, because gene therapies have the potential to treat many neurological disorders, researchers are working on them. Some of the fundamental experimental approaches for gene therapy are listed in Table 1. Even while there is no concrete evidence that gene therapy may entirely cure KAND, the preliminary findings from research trials enable the researchers to focus more on creating a treatment plan. These treatments will target the genes that cause the condition as well as the neurotrophic factors that support the healthy function and survival of the neuronal cell [52]. The use of nanoparticles, engineered microRNA, plasmid transfection, viral vector design, polymer-mediated gene delivery, clustered regularly interspaced short palindromic repeats (CRISPR)-based therapeutics, and other technologies has advanced this field [52]. Because surgical treatment cannot cure KAND and there is currently no approved standard pharmacological treatment, gene-based therapeutics are crucial [53]. By fully comprehending the pathophysiology of the disease and then treating it at the molecular level, these technologies also have the advantage of repairing genes and treating disorders that are not at all treatable by utilizing conventional medical procedures [54,55]. 

The choice of a gene transfer vector is crucial because it directly affects how effective the treatment will be. The selection process takes into account a number of variables, including affinity and blood-brain barrier (BBB) crossing capability. Adenovirus (Ad), Herpes-Simplex virus (HSV), Lentivirus (LV), and recombinant Adeno-Associated virus (rAAV) are some of the most often employed viral vectors [56]. Even though all these methods exist to treat KAND, there is no conclusive clinical evidence that they can also be used to treat KRDs. The effectiveness percentage is still unknown. 

## 7. *De Novo* Variations in the KIF1A Gene

*De novo* variants are primarily seen in patients who also have comorbid conditions like cognitive impairment, muscle stiffness, or optic nerve atrophy. These conditions co-occur with clinical symptoms caused by recessive mutations in the *KIF1A* gene, making them more harmful. The majority of mutations are found in the motor domain and are easily anticipated since they have an impact on the protein’s ability to operate normally as a motor because of the change in the original structure [60]. Only a limited amount of information is known regarding *de novo* mutations because there haven’t been many reports on these mutations published (Table 2). The missense mutations are observed at the c.296C > T/p.Thr99Met location of the KIF1A gene, which can affect the amino acid produced which will directly have an impact on the protein functions [17]. The prediction algorithms used are Scale Invariant Feature Transform (SIFT) [61], Polymorphism Phenotyping v2 (PolyPhen-2) [62], and PANTHER [63]. 

The following clinical manifestations are observed in people with *de novo* mutations in the motor domain:

- Intellectual disability—delay in cognitive development occurs in all cases
- Cerebellar atrophy—diagnosed using magnetic resonance imaging
- Optic nerve atrophy
- Spastic paraplegia—mainly affecting lower limbs
- Peripheral neuropathy [14]

## 8. Relation between KIF1A Variants and HSP

### 8.1. HSP

An uncommon neurological condition called HSP causes stiffness or wasting of the bladder or lower limbs [37]. It is caused by X chromosome-linked inheritance patterns, autosomal dominant and recessive mutations, as well as other factors that are categorized in OMIM [64]. Autosomal dominant paraplegia HSP subtype SPG30 is a result of homozygous missense variants, SPG7 and SPG11 in the *KIF1A* gene whereas the dominant form of HSP is observed due to variation in the ‘SPAST’ gene [38,65,66]. HSP can manifest basically in the form of spasticity and weakness in the patient. Although they may also experience hyperuricemia, these patients’ life expectancies are unaffected. In more severe cases of HSP, peripheral and optic neuropathy as well as mental impairment may also be present. Currently, 79 specific and fixed positions are located in chromosomes with 61 corresponding genes that have a link to HSP condition [37]. 

The following types are the commonly recognized HSP disorders:

- Spastic paraplegia + Peripheral motor neuropathy + Distal wasting
- Spastic paraplegia + Cognitive impairment
- Spastic paraplegia + Ataxia
- Spastic paraplegia + Neuroimaging abnormality
- Spastic paraplegia + Additional neurologic + Systemic abnormalities [66].

### 8.2. KIF1A Variants and Spastic Paraplegia

KIF1A mutations are seen in three regions; the motor domain, regulatory region, and cargo binding region and these are mainly responsible for the development of SPG30. Specific mutations affect the gene function in a specific manner. These changes in the functions will ultimately result in the mislocalization of cellular cargoes, i.e., it will make the KIF1A protein unable to regulate its motility and subsequently, it fails to bind to the cargo. The severity of SPG30 depends on to what extent the *KIF1A* gene has undergone mutations [15]. The loss of functioning in the motor domain of KIF1A can affect the structural domain which is essential for various functions such as hydrolyzing ATP, providing mechanical force, and MT-binding (loop L8). Examples of these can be mutations residing in Switch I represented as R216C and Switch II which is represented as E253K and mutations that cause destabilization of loop L8 [66,67]. Switch II is better understood as E253K and ATP-binding cassette (ABC), that is the ATP-binding cassette mutant which drastically slows down the motility of this motor protein resulting in the inability to move to the distal portion of the neuronal axon.

The variants which result in the loss of functionality of the gene located outside the kinesin domain of the motor region lead to a defect in normal functioning that causes a problem known as functional intolerance [68,69]. Interestingly, a gain of function has also been observed in SPG30. Making use of a single-molecule assay procedure, Chiba et al. reported V8M, R350G, and A255V, the three KIF1A mutants casual in SPG30 had higher rates of settling on MTs and had higher velocity as compared to wild type (WT) KIF1A, indicating that excessive cargo accumulation can be harmful. In cohort studies done by Maartje Pennings et al., 20 heterozygous KIF1A variants were reported by clinical exome sequencing and the resulting SPG due to KIF1A was pure. It was observed that phenotypic differences in the KIF1A-related diseases may be due to different levels of impairment in transport. Parental testing done by the team revealed the deletion of chr2q37 in a few families. *KIF1A* gene is localized in the cytogenic 2q37.3 band and microdeletion of chromosome 2q37 is deleted in patients suffering from 2q37 microdeletion syndrome that can be observed by intellectual disability, brachydactyly, weight gain, hypotonia, characteristic facial features, autism, and epilepsy [37,67]. Eleven of the 20 variants reported in the studies done by Maartje Pennings et al. were found to be missense variants located at the motor domain that cause dominant SPG. The rest nine variants detected outside the motor domain included variants that showed loss of functionality of gene (some were *de novo* occurrences) and chr2q37 deletion which indicates that loss of function variants has the ability to cause autosomal dominant SPG [37]. 

In another study done by Stephan Klebe et al., using targeted NGS, p.R350G variant was identified, which has a direct effect on amino acid in the motor domain of kinesin 1A, and surprisingly this variant was found to be compatible with phenotype expressed by HSP patients. In the same studies, whole-genome genotyping done in a Palestinian family revealed that there is the presence of a unique homozygous c.756>T [p. Ala255Val] mutation that caused the phenotypic symptoms. Studies have shown that the nature of mutations could help scientists to foresee the phenotype expressed. Non-sense mutations which can lead to complete loss of functionality of the protein can cause significant clinical manifestations in the peripheral nervous system (PNS), as peripheral neuropathy is common in more than 60% of the SPG30 patients [22,70,71].

## 9. KIF1A and Brain Atrophy

Homozygous mutation in KIF1A is one of the main reasons for the rare hereditary sensory and autonomic neuropathy (HSAN) and HSP, but experiments that were done in vitro suggest that homozygous mutations influence the transport through synaptic vesicles and can lead to axon degeneration [22,66]. For example, in a child, a pathogenic variant, p.T99M *de novo* variation that causes cerebellar atrophy was reported, indicating these mutations may alter the neuronal function by disabling kinesin-mediated cargo transport. It has been observed that the homozygous inactivation KIF1A gene in mice can cause severe motor as well as sensory disturbances [72]. In studies done by Sahar Esmaeeli Nieh et al. [6] on 6 different patients, five *de novo* mutations were identified out of which two patients were observed who had *de novo* c.296C>T change that contained a substitution of threonine to methionine also represented as T99M [17]. Mutations like p.E253K also represented as c.757G>A and p.R316W were reported in the other two patients tested. The rest two patients had changes in the amino acid residues that were again getting mutated to form a third amino acid variant [19]. All these mutations were identified within a conserved region of the motor domain and they have the capacity to cause damage by using PolyPhen-2 [73,74]. 

In another study done by Chihiro Ohba et al., 5 missense mutations were found in five patients and were confirmed by Sanger sequencing to be *de novo* events. Magnetic resonance imaging done by the same team noticed that the patients had some difficulties in the gait along with exaggerated reflexes from locations such as deep tendons and in a few patients, cerebellar atrophy was observed. All *de novo* mutations observed during this study are located in the motor domain which mostly affects motor function. In this study, all the mutations observed were identified in the motor domain. The mutation p.Arg316Trp had been previously reported [72], at the same time Arg254, Arg307, and Arg307 were found on n loop L11. The α5 helix that helps to induce phosphate release during the hydrolysis of adenosine triphosphate molecule and facilitates KIF1A protein to bind onto MTs was also found to be mutated in some individuals [19,69,75]. The mutations can exhibit some unique actions on the structures present near them such as the p.Glu253Lys mutation adjacent to Arg254 can suppress γ-phosphate release [19], while p.Arg316Trp mutation disrupts the stability of loop 8 which forms a bond with the MT [69]. 

### 9.1. NESCAV Syndrome

NESCAV syndrome (NESCAVS), also referred to as autosomal dominant 9 or intellectual disability, is a neurodegenerative disorder characterized by global development delay with delayed walking or difficulty in walking due to spasticity in the lower limbs leading to loss of independent ambulation [2]. Some of the clinical features include optic nerve atrophy and varying degrees of brain atrophy, microcephaly, joint contractures, kyphosis, clubfoot, spasticity, and cerebellar atrophy [76]. It has been observed that NESCAVS is caused due to *de novo* heterozygous T99M mutation in the *KIF1A* gene. This study was done on an 8-year-old Japanese boy having axial hypotonia, peripheral spasticity, and global development delay with additional clinical manifestations like growth hormone deficiency, neurogenic bladder, and constipation [77]. In a few other studies involving unrelated patients, other manifestations like cortical visual impairment, optic neuropathy, movement disorders [6], hyperreflexia, hypermetrophic astigmatism, oculomotor apraxia, and distal muscle weakness [60]. Hamdan et al. (2011), identified a *de novo* missense mutation in the *KIF1A* gene in a patient with NESCAVS. In his study, he inserted a KIF1A MD-EGFP fusion construct into the hippocampal neurons present in rats and showed that the distal localization gets greatly reduced in neurites carrying the T99M mutation which leads to increased accumulation [17]. These mutations are found by whole-genome sequencing which can be later confirmed by sanger sequencing [60].

### 9.2. PEHO Syndrome [OMIM No. 260565]

PEHO syndrome characterized by progressive encephalopathy along with edema, and hypsarrhythmia is a rare neurodegenerative disease which leads to total loss of granules in the neurons resulting in an extreme condition of cerebellar atrophy [78]. This condition was first reported in 14 Finnish families in the year 1991. The basis for the diagnosis of PEHO syndrome has been put out by Somer et al. [79] who recognized the necessary features of this condition, i.e., jerking along with spasms, brain atrophy on neuroimaging studies, especially in the cerebellum and few regions of the brain stem with mild supratentorial atrophy [80]. In studies done by Sylvie Langlois et al., which involved the genomic study of patients with PEHO syndrome is being described; nine candidate genes were identified using trio WES out of which eight genes were heterozygous variants and a gene was *de novo* variant. The missense variant, p.(T99M) in KIF1A residing in chromosome number 2 is considered pathogenic [81]. Sanger sequencing was also carried out on the female patient and the unaffected parents and it was proved that the patient was heterozygous for the variant. Before this study was made, 24 patients had been reported with *de novo* heterozygous variants affecting the motor domain of KIF1A protein and the functional impact of these variants was demonstrated by Lee et al. The main clinical features reported were moderate to severe developmental delay, cerebellar atrophy, optic nerve atrophy, progressive spasticity affecting lower limbs, and peripheral neuropathy [19].

## 10. KIF1A and Autism Spectrum Disorder [ASD]

ASD also known as autism is a type of neurodevelopmental disorder in which the patients show a deficit in communication as well as the processing of language and expression of thoughts. This disorder directly or indirectly can influence the social life of the patient to a great extent. There are several genes implicated in ASD but approximately 10–15% of cases are due to mutations in a single gene [82]. Reports suggest that the patients exhibiting complex phenotypes are characterized by axonal neuropathy, spasticity, and majorly ASD. The genetic examination of these patients revealed about 21,683 variants in the coding regions [83]. Another study reports that there is a link between the KIF1A mutations and autism and is normally characterized by other neurological conditions like sensory disturbance, hyperactivity, spastic paraplegia, and epilepsy. Normally the c.38 g>A [R13H] mutation exhibits autism and hyperactivity, but in some special cases, all the neurological symptoms are exhibited by c.37C>T (p.R13C) which is a *de novo* mutation [84]. 

In the majority of the research studies done on KAND, the peripheral blood is used as the sample and the DNA is extracted and the gene sequencing is mainly done by WES technology. There are modern tools also available for predicting the structure such as the SWISS-MODEL and Mutation Tester that utilize a different strategy for reporting [85]. If a patient is found to have harmful variants such as c.664A>C (p. Asn222His), a type of *de novo* variant, it is suggested that the patient is at a higher risk of getting ASD [85]. Not only this, one interesting thing to be noted is that along with KIF1A mutations, the mutations on the HUWE1 gene have also led to the expression of ASD and other conditions like epilepsy. This is mainly due to the 22q11.2 duplication (a penetrant copy number variant) [86]. These studies suggest that ASD is having a close association with KAND.

## 11. Recent Studies in the Field of KAND

Transport of cargo is very much important as far as a cell is concerned. If proper translocation does not take place, the cargo can get accumulated and lead to cell necrosis. The transport of cargo is done by three major methods:

- Regulation of motor ATPase activity by the process of autoinduction.
- Regulation of cargo adaptors
- Modifications on the cytoskeletal tract.

There have been tremendous efforts done by a lot of scientists to discover the in vivo functioning of each part of the *KIF1A* gene and its protein. Recent advancements in technology have led to the discovery of newer models such as the DNA origami scaffold model which provides a much more precise picture of what is happening *in vivo*. We have included some of the latest discoveries in KAND in this paper. In a few studies done recently, it was proved that a more bound linker will allow the cargo motors to attach to the MT track. It was found that KIF1A, KIF13B, and KIF16 regulate the parts of the KIF1A protein, especially the coiled domain [30]. Another study shows that the kinesin-3 monomers can be multimerized which results in the transport of cargo [87]. Most of the studies done on the regulation of motor domains are normally carried out using the pure components of proteins, particularly in the motor domain but there are a few exemptions to be noted here such as the functioning of two opposite domains in the protein [88,89,90,91,92]. 

When transport of the cargo does not take place in a neuronal cell, the kinesin motors especially the kinesin-3 subfamily adopt the mechanism of autoinhibited conformation. In such a case, the UNC-104KIF1A gets activated automatically. To date, the mechanism of activation is not understood fully. This condition leads to the enhancement in cargo transport in the form of vesicles in the cell. This also explains to us the cause of motor hyperactivation associated with this disease [93]. In some cases, it is reported that there can be a disruption of motor domain/CC1 domain-mediated autoinhibition due to the actions of dominant suppressors. To be more specific, the mutations at C184 can disrupt the inter-domain packing, while if the mutation takes place at the G421 then there will be a sudden turn between the CC1a and CC1b that can indirectly lead to interference in packing [12,94,95,96,97]. 

## 12. Gene Therapy

In order for the transgene to integrate with the host DNA (retrovirus) and make up for the defective gene’s lack of expression, the transgene must be introduced into the target cells through gene transfer therapy. Scientists have developed a wide range of carriers for the successful delivery of genes to their targets and the majority of them comprise of plasmid DNA and oligonucleotides [98]. Despite the fact that gene therapy overcomes the difficulties associated with conventional treatment, it is not without drawbacks. They include the high cost of gene therapy, which restricts its use, ethical concerns about changes made to the germline, immune rejection of the transferred gene, and the route of administration [96]. Due to their low pathogenicity, cellular tropism, replication incompetency, and simplicity of manipulation, LV and AAVs are being investigated as delivery modalities for the introduction of transgenes in clinical trials. A naturally occurring serotype of AAV called AAV9 has the capacity to penetrate the BBB and target neurons, astrocytes, and microglia in the brain. The capsid proteins of these serotypes distinguish them and help determine the corresponding cellular tropism [99]. 

Due to its cell-specific transduction abilities, rAAV9 is the most recommended CNS delivery technique for neurological diseases. Both dividing and non-dividing cells can be transduced by rAAVs [98]. The three most effective gene editing techniques used for modifying cellular DNA at the native locus are CRISPR and CRISPR-associated (Cas) proteins, transcription activator-like effector nucleases (TALENs), and zinc finger nucleotides (ZFN). ZFNs are the first programmable nucleases that can cleave particular regions of DNA using an altered *Fok*l endonuclease to change the way double-stranded breaks are repaired in DNA [100]. Target gene alterations can be carried out using TALENs, which can recognize random target sequences. TALENs merge *Fok*l endonuclease with transcription activator-like effectors (TALEs) modular DNA binding domain [101].

The foundation of gene editing techniques is the introduction of genomic breaks and the precise allocation of these breaks by nuclease enzymes. Genome editing depends on two biological pathways: non-homologous end joining (NHEJ) and homology-directed repair (HDR) [102]. While NHEJ is frequently observed in cells that are not dividing, such as neurons, HDR occurs across all cell cycle phases. The NHEJ repair process has been used by researchers to support gene editing techniques. Several unique gene editing techniques have been created thus far for non-dividing cells, including, HITI (homology independent targeted integration) [103], HITI-based SATI approach (Single homology Arm donor mediated Intron-Targeting Integration) [104], CRISPR Prime editing [105], HMEJ (homology mediated end joining) [106], vSLENDR (virus-mediated single-cell labelling of endogenous proteins via HDR) [107], PITCh (precise integration into targeted chromosome) [108].

A genomic technique known as whole exome sequencing (WES) is used to sequence the protein-coding sections of genomic DNA (exons) and find the causative mutations that lead to specific genetic disorders [109,110]. WES has been extensively utilized to diagnose KRDs [48,70]. The use of gene therapy to treat neurogenetic disorders has risen dramatically over the past several years, with KAND increasingly displacing more traditional approaches. As previously stated, there is no known treatment for KAND. The development of gene therapy offers hope for the existing therapeutic approaches. Gene editing tools now make it possible to change genes, and there is a chance that the conditions could be reversed. However, there are several drawbacks to gene therapy, such as inserted gene over- or under-expression, vector capacity to carry the gene, and mutant gene product attacking the wild-type allele. The constraints of gene therapy have been overcome by the emergence of gene editing tools like CRISPR Cas9.

## 13. Conclusions

In this paper, we addressed all the available information, recent studies, and newer advancements concerning the KAND. The main cause of this disease is the mutations that take place in the *KIF1A* gene which can result in loss of function or gain of mutated functions. These mutations directly result in the mis-delivery of essential cargo transported inside the neurons. These cargoes play a very important role in neuronal growth, differentiation, and survival. Another problem is that there can be different phenotypic expressions even for the same mutation in the gene making the diagnosis difficult even with the help of expensive testing. It was observed that there can be two different forms of KIF1A-related disorders which are the autosomal dominant forms as well as autosomal recessive forms. One such condition is spastic paraplegia, which has been discussed earlier in this paper. Spastic paraplegia can be pure when symptoms are confined to stiffness in the legs and bladder. When these symptoms are accompanied by other neurological disturbances, then it is termed as complicated. KIF1A mutations are also linked to brain atrophy, encephalopathy, PEHO syndrome and autism spectrum disorder and all the disorders were observed to occur due to *de novo* variations in the *KIF1A* gene. Very few reports were available regarding the *de novo* variation, which has been mentioned in the article.

There are various organizations and foundations that provide information and spread awareness about KAND. Some are governmental while others are privately funded organizations. These organizations consist of the patients, family members of the patient as well as the physicians, clinicians, research scholars, and other paramedical staff. Although this disorder is rare and there are still many research gaps in the field of KAND in neuroscience, the possibility of the development of a new drug or an active chemical moiety cannot be predicted as of now. To date, there is no existing cure that guarantees the recovery of the patient, but we expect that newer advancements in neuroscience can enhance the treatment and management of KAND. A better understanding of the in vivo functioning of the motor domain, the part where most of the mutations take place can give us a lead to improve the existing difficulties faced by the patients. Newer models like the DNA origami scaffold model are used by scientists to provide more information on what is happening *in vivo*. Newer technologies such as gene therapy have the potential to pave way for advanced therapies and thereby increasing the quality of life of the patients.

## Figures and Tables

**Table 1:** The fundamental experimental approach for gene therapy used four theoretical modes of action [56].

Sl no.	Modes of Action	Specification
1.	Gene replacement [57]	This is done when the disease is caused due to the loss of functionality of the gene.
2.	Gene knockdown [58]	This is employed when a function has been toxically increased or when gene metabolites or gene products have accumulated.
3.	Pro-survival or symptomatic gene therapy [56]	Here the pathological condition is reversed by using a pro-survival gene that is non-specific in nature.
4.	Cell suicide gene therapy [59]	This is typically thought of as the last option. This is primarily used in cancer treatment, where it is necessary to eradicate malignant cells. In the case of KAND, this method’s application is constrained.

**Table 2:** Various parts of the KIF1A protein susceptible to mutation [26,27,28,29].

Sl no.	Domains	Proteins under Mutations.
1.	Motor domain	p.S58L p.G102D p.V144F p.R167C p.A202P p.S215R p.R216P p.L249Q p.E253K p.R316W
2.	Forkhead-associated domain	p.L947Rfs*4
3.	Pleckstrin homology	p.S1758Qfs*7

## Data Availability

The data sets used, analyzed, and reviewed were collected from the corresponding authors and online research databases.

## Abbreviations

ABC	ATP binding cassette
ASD	autism spectrum disorder
CRISPR	Clustered regularly interspaced short palindromic repeats
DNA	Deoxyribonucleic acid
DRD1	Dopamine receptor D1
FHA	Forkhead associated domain
HSAN	Hereditary sensory and autonomic neuropathy
HSP	Hereditary spastic paraplegia
HSV	Herpes simplex virus
KAND	KIF1A associated neurological disorders
KRD	KIF1A related disorders
LV	Lenti virus
OMIM	Online Mendelian Inheritance in Man
PEHO	Progressive encephalopathy with oedema, Hypsarrhythmia and optic atrophy
PH	Pleckstrin homology
rAAV	recombinant Adenome associated virus
SIFT	Scale in variant feature transform
SPG30	Spastic paraplegia 30
WES	Whole exome sequencing

## References

[1] Roda R.H., Schindler A.B., Blackstone C. (2017). Multigeneration family with dominant SPG30 hereditary spastic paraplegia. Ann. Clin. Transl. Neurol..

[2] Nemani T., Steel D., Kaliakatsos M., DeVile C., Ververi A., Scott R., Getov S., Sudhakar S., Male A., Mankad K. (2020). *KIF1A* -related disorders in children: A wide spectrum of central and peripheral nervous system involvement. J. Peripher. Nerv. Syst..

[3] Boyle L., Rao L., Kaur S., Fan X., Mebane C., Hamm L., Thornton A., Ahrendsen J.T., Anderson M.P., Christodoulou J. (2021). Genotype and defects in microtubule-based motility correlate with clinical severity in KIF1A-associated neurological disorder. Hum. Genet. Genom. Adv..

[4] Di Fabio R., Comanducci G., Piccolo F., Santorelli F.M., DE Berardinis T., Tessa A., Sabatini U., Pierelli F., Casali C. (2012). Cerebellar Atrophy in Congenital Fibrosis of the Extraocular Muscles Type 1. Cerebellum.

[5] Baptista F., Pinto M.J., Elvas F., Almeida R., Ambrósio A.F. (2013). Diabetes Alters KIF1A and KIF5B Motor Proteins in the Hippocampus. PLoS ONE.

[6] Nieh S.E., Madou M.R.Z., Sirajuddin M., Fregeau B., McKnight D., Lexa K., Strober J., Spaeth C., Hallinan B.E., Smaoui N. (2015). De novo mutations in KIF1A cause progressive encephalopathy and brain atrophy. Ann. Clin. Transl. Neurol..

[7] Montenegro-Garreaud X., Hansen A.W., Khayat M.M., Chander V., Grochowski C.M., Jiang Y., Li H., Mitani T., Kessler E., Jayaseelan J. (2020). Phenotypic expansion in *KIF1A*-related dominant disorders: A description of novel variants and review of published cases. Hum. Mutat..

[8] Aguilera C., Hümmer S., Masanas M., Gabau E., Guitart M., Jeyaprakash A.A., Segura M.F., Santamaria A., Ruiz A. (2021). The Novel KIF1A Missense Variant (R169T) Strongly Reduces Microtubule Stimulated ATPase Activity and Is Associated with NESCAV Syndrome. Front. Neurosci..

[9] Bharat V., Siebrecht M., Burk K., Ahmed S., Reissner C., Kohansal-Nodehi M., Steubler V., Zweckstetter M., Ting J.T., Dean C. (2017). Capture of Dense Core Vesicles at Synapses by JNK-Dependent Phosphorylation of Synaptotagmin-4. Cell Rep..

[10] Okada Y., Yamazaki H., Sekine-Aizawa Y., Hirokawa N. (1995). The neuron-specific kinesin superfamily protein KIF1A is a uniqye monomeric motor for anterograde axonal transport of synaptic vesicle precursors. Cell.

[11] Hammond J., Cai D., Blasius T.L., Li Z., Jiang Y., Jih G.T., Meyhofer E., Verhey K.J. (2009). Mammalian Kinesin-3 Motors Are Dimeric In Vivo and Move by Processive Motility upon Release of Autoinhibition. PLOS Biol..

[12] Klopfenstein D.R., Tomishige M., Stuurman N., Vale R.D. (2002). Role of Phosphatidylinositol(4,5)bisphosphate Organization in Membrane Transport by the Unc104 Kinesin Motor. Cell.

[13] Huo L., Yue Y., Ren J., Yu J., Liu J., Yu Y., Ye F., Xu T., Zhang M., Feng W. (2012). The CC1-FHA Tandem as a Central Hub for Controlling the Dimerization and Activation of Kinesin-3 KIF1A. Structure.

[14] Lee J.-R., Shin H., Ko J., Choi J., Lee H., Kim E. (2003). Characterization of the Movement of the Kinesin Motor KIF1A in Living Cultured Neurons. J. Biol. Chem..

[15] Gabrych D.R., Lau V.Z., Niwa S., Silverman M.A. (2019). Going Too Far Is the Same as Falling Short†: Kinesin-3 Family Members in Hereditary Spastic Paraplegia. Front. Cell. Neurosci..

[16] Citterio A., Arnoldi A., Panzeri E., Merlini L., D’Angelo M.G., Musumeci O., Toscano A., Bondi A., Martinuzzi A., Bresolin N. (2015). Variants in KIF1A gene in dominant and sporadic forms of hereditary spastic paraparesis. J. Neurol..

[17] Hamdan F.F., Gauthier J., Araki Y., Lin D.-T., Yoshizawa Y., Higashi K., Park A.-R., Spiegelman D., Dobrzeniecka S., Piton A. (2011). Excess of De Novo Deleterious Mutations in Genes Associated with Glutamatergic Systems in Nonsyndromic Intellectual Disability. Am. J. Hum. Genet..

[18] Ylikallio E., Kim D., Isohanni P., Auranen M., Kim E., Lönnqvist T., Tyynismaa H. (2015). Dominant transmission of de novo KIF1A motor domain variant underlying pure spastic paraplegia. Eur. J. Hum. Genet..

[19] Lee J.-R., Srour M., Kim D., Hamdan F.F., Lim S.-H., Brunel-Guitton C., Décarie J.-C., Rossignol E., Mitchell G.A., Schreiber A. (2015). De Novo Mutations in the Motor Domain of KIF1A Cause Cognitive Impairment, Spastic Paraparesis, Axonal Neuropathy, and Cerebellar Atrophy. Hum. Mutat..

[20] Niwa S., Lipton D.M., Morikawa M., Zhao C., Hirokawa N., Lu H., Shen K. (2016). Autoinhibition of a Neuronal Kinesin UNC-104/KIF1A Regulates the Size and Density of Synapses. Cell Rep..

[21] Iqbal Z., Rydning S.L., Wedding I.M., Koht J., Pihlstrøm L., Rengmark A.H., Henriksen S.P., Tallaksen C.M.E., Toft M. (2017). Targeted high throughput sequencing in hereditary ataxia and spastic paraplegia. PLoS ONE.

[22] Rivière J.-B., Ramalingam S., Lavastre V., Shekarabi M., Holbert S., Lafontaine J., Srour M., Merner N., Rochefort D., Hince P. (2011). KIF1A, an Axonal Transporter of Synaptic Vesicles, Is Mutated in Hereditary Sensory and Autonomic Neuropathy Type 2. Am. J. Hum. Genet..

[23] Chiba K., Takahashi H., Chen M., Obinata H., Arai S., Hashimoto K., Oda T., McKenney R.J., Niwa S. (2019). Disease-associated mutations hyperactivate KIF1A motility and anterograde axonal transport of synaptic vesicle precursors. Proc. Natl. Acad. Sci. USA.

[24] Pyrpassopoulos S., Gicking A.M., Zaniewski T.M., Hancock W.O., Ostap E.M. (2023). KIF1A is kinetically tuned to be a super-engaging motor under hindering loads. Biophys. Comput. Biol..

[25] Hirokawa N., Noda Y., Tanaka Y., Niwa S. (2009). Kinesin superfamily motor proteins and intracellular transport. Nat. Rev. Mol. Cell Biol..

[26] Budaitis B.G., Jariwala S., Rao L., Yue Y., Sept D., Verhey K.J., Gennerich A. (2021). Pathogenic mutations in the kinesin-3 motor KIF1A diminish force generation and movement through allosteric mechanisms. J. Cell Biol..

[27] Okada Y., Hirokawa N. (1999). A Processive Single-Headed Motor: Kinesin Superfamily Protein KIF1A. Science.

[28] Soppina V., Verhey K.J. (2014). The family-specific K-loop influences the microtubule on-rate but not the superprocessivity of kinesin-3 motors. Mol. Biol. Cell.

[29] Okada Y., Hirokawa N. (2000). Mechanism of the single-headed processivity: Diffusional anchoring between the K-loop of kinesin and the C terminus of tubulin. Proc. Natl. Acad. Sci. USA.

[30] Soppina V., Norris S.R., Dizaji A.S., Kortus M., Veatch S., Peckham M., Verhey K.J. (2014). Dimerization of mammalian kinesin-3 motors results in superprocessive motion. Proc. Natl. Acad. Sci. USA.

[31] Alushin G.M., Lander G.C., Kellogg E.H., Zhang R., Baker D., Nogales E. (2014). High-Resolution Microtubule Structures Reveal the Structural Transitions in αβ-Tubulin upon GTP Hydrolysis. Cell.

[32] Zhang R., LaFrance B., Nogales E. (2018). Separating the effects of nucleotide and EB binding on microtubule structure. Proc. Natl. Acad. Sci. USA.

[33] Zhang R., Alushin G.M., Brown A., Nogales E. (2015). Mechanistic Origin of Microtubule Dynamic Instability and Its Modulation by EB Proteins. Cell.

[34] Montemurro N., Ricciardi L., Scerrati A., Ippolito G., Lofrese G., Trungu S., Stoccoro A. (2022). The Potential Role of Dysregulated miRNAs in Adolescent Idiopathic Scoliosis and 22q11.2 Deletion Syndrome. J. Pers. Med..

[35] Anazawa Y., Kita T., Iguchi R., Hayashi K., Niwa S. (2022). De novo mutations in KIF1A-associated neuronal disorder (KAND) dominant-negatively inhibit motor activity and axonal transport of synaptic vesicle precursors. Proc. Natl. Acad. Sci..

[36] Lumpkins C. KIF1A-Related Disorder. NORD (National Organization for Rare Disorders). https://rarediseases.org/rare-diseases/kif1a-related-disorder/.

[37] Pennings M., Schouten M.I., van Gaalen J., Meijer R.P.P., de Bot S.T., Kriek M., Saris C.G.J., Berg L.H.V.D., van Es M.A., Zuidgeest D.M.H. (2019). KIF1A variants are a frequent cause of autosomal dominant hereditary spastic paraplegia. Eur. J. Hum. Genet..

[38] Klebe S., Lossos A., Azzedine H., Mundwiller E., Sheffer R., Gaussen M., Marelli C., Nawara M., Carpentier W., Meyer V. (2012). KIF1A missense mutations in SPG30, an autosomal recessive spastic paraplegia: Distinct phenotypes according to the nature of the mutations. Eur. J. Hum. Genet..

[39] McEntagart M.E., Reid S.L., Irtthum A., Douglas J.B., Eyre K.E.D., Donaghy M.J., Anderson N.E., Rahman N. (2005). Confirmation of a hereditary motor and sensory neuropathy IIC locus at chromosome 12q23-q24. Ann. Neurol..

[40] Hedera P., Adam M., Everman D., Mirzaa G., Pagon R., Wallace S., Bean L.J., Gripp K., Amemiya A. (1993). Hereditary Spastic Paraplegia Overview. GeneReviews®.

[41] Hebbar M., Shukla A., Nampoothiri S., Bielas S., Girisha K.M. (2018). Locus and allelic heterogeneity in five families with hereditary spastic paraplegia. J. Hum. Genet..

[42] Fink J.K., Rosenberg R.N., Pascual J.M. (2015). Chapter 77—Hereditary Spastic Paraplegia. Rosenberg’s Molecular and Genetic Basis of Neurological and Psychiatric Disease.

[43] Rauch A., Hoyer J., Guth S., Zweier C., Kraus C., Becker C., Zenker M., Hüffmeier U., Thiel C., Rüschendorf F. (2006). Diagnostic yield of various genetic approaches in patients with unexplained developmental delay or mental retardation. Am. J. Med. Genet. Part A.

[44] Miller D.T., Adam M.P., Aradhya S., Biesecker L.G., Brothman A.R., Carter N.P., Church D.M., Crolla J.A., Eichler E.E., Epstein C.J. (2010). Consensus Statement: Chromosomal Microarray Is a First-Tier Clinical Diagnostic Test for Individuals with Developmental Disabilities or Congenital Anomalies. Am. J. Hum. Genet..

[45] Costain G., Cordeiro D., Matviychuk D., Mercimek-Andrews S. (2019). Clinical Application of Targeted Next-Generation Sequencing Panels and Whole Exome Sequencing in Childhood Epilepsy. Neuroscience.

[46] Demos M., Guella I., DeGuzman C., McKenzie M.B., Buerki S.E., Evans D.M., Toyota E.B., Boelman C., Huh L.L., Datta A. (2019). Diagnostic Yield and Treatment Impact of Targeted Exome Sequencing in Early-Onset Epilepsy. Front. Neurol..

[47] Watson E., Davis R., Sue C.M. (2020). New diagnostic pathways for mitochondrial disease. J. Transl. Genet. Genom..

[48] Lee H., Chi C., Tsai C. (2020). Diagnostic yield and treatment impact of whole-genome sequencing in paediatric neurological disorders. Dev. Med. Child Neurol..

[49] (2022). KIF1A. Wikipedia. https://en.wikipedia.org/w/index.php?title=KIF1A&oldid=1077108149.

[50] Barbitoff Y.A., Polev D.E., Glotov A.S., Serebryakova E.A., Shcherbakova I.V., Kiselev A.M., Kostareva A.A., Glotov O.S., Predeus A.V. (2020). Systematic dissection of biases in whole-exome and whole-genome sequencing reveals major determinants of coding sequence coverage. Sci. Rep..

[51] Clark M.M., Stark Z., Farnaes L., Tan T.Y., White S.M., Dimmock D., Kingsmore S.F. (2018). Meta-analysis of the diagnostic and clinical utility of genome and exome sequencing and chromosomal microarray in children with suspected genetic diseases. npj Genom. Med..

[52] Pena S.A., Iyengar R., Eshraghi R.S., Bencie N., Mittal J., Aljohani A., Mittal R., Eshraghi A.A. (2019). Gene therapy for neurological disorders: Challenges and recent advancements. J. Drug Target..

[53] Simonato M., Bennett J., Boulis N.M., Castro M.G., Fink D.J., Goins W.F., Gray S.J., Lowenstein P.R., Vandenberghe L.H., Wilson T.J. (2013). Progress in gene therapy for neurological disorders. Nat. Rev. Neurol..

[54] Puranik N., Yadav D., Chauhan P.S., Kwak M., Jin J.-O. (2021). Exploring the Role of Gene Therapy for Neurological Disorders. Curr. Gene Ther..

[55] Choong C.-J., Baba K., Mochizuki H. (2015). Gene therapy for neurological disorders. Expert Opin. Biol. Ther..

[56] Manfredsson F.P., Mandel R.J. (2010). Development of gene therapy for neurological disorders. Discov. Med..

[57] Khan S.M., Bennett J.P. (2004). Development of Mitochondrial Gene Replacement Therapy. J. Bioenerg. Biomembr..

[58] Shan G. (2010). RNA interference as a gene knockdown technique. Int. J. Biochem. Cell Biol..

[59] Bonini C., Bondanza A., Perna S.K., Kaneko S., Traversari C., Ciceri F., Bordignon C. (2007). The Suicide Gene Therapy Challenge: How to Improve a Successful Gene Therapy Approach. Mol. Ther..

[60] Ohba C., Haginoya K., Osaka H., Kubota K., Ishiyama A., Hiraide T., Komaki H., Sasaki M., Miyatake S., Nakashima M. (2015). De novo KIF1A mutations cause intellectual deficit, cerebellar atrophy, lower limb spasticity and visual disturbance. J. Hum. Genet..

[61] Ng P.C., Henikoff S. (2003). SIFT: Predicting amino acid changes that affect protein function. Nucleic Acids Res..

[62] Sunyaev S. (2001). Prediction of deleterious human alleles. Hum. Mol. Genet..

[63] Thomas P.D., Kejariwal A., Guo N., Mi H., Campbell M.J., Muruganujan A., Lazareva-Ulitsky B. (2006). Applications for protein sequence-function evolution data: mRNA/protein expression analysis and coding SNP scoring tools. Nucleic Acids Res..

[64] Giudice T.L., Lombardi F., Santorelli F.M., Kawarai T., Orlacchio A. (2014). Hereditary spastic paraplegia: Clinical-genetic characteristics and evolving molecular mechanisms. Exp. Neurol..

[65] Klebe S., Azzedine H., Durr A., Bastien P., Bouslam N., Elleuch N., Forlani S., Charon C., Koenig M., Melki J. (2006). Autosomal recessive spastic paraplegia (SPG30) with mild ataxia and sensory neuropathy maps to chromosome 2q37.3. Brain.

[66] Fink J. (2014). Hereditary Spastic Paraplegia: Clinical Principles and Genetic Advances. Semin. Neurol..

[67] Doherty E.S., Lacbawan F.L., Adam M., Everman D., Mirzaa G., Pagon R., Wallace S., Bean L.J., Gripp K., Amemiya A. (1993). 2q37 Microdeletion Syndrome—Retired Chapter, For Historical Reference Only. GeneReviews®.

[68] Cheon C.K., Lim S.-H., Kim Y.-M., Kim D., Lee N.-Y., Yoon T.-S., Kim N.-S., Kim E., Lee J.-R. (2017). Autosomal dominant transmission of complicated hereditary spastic paraplegia due to a dominant negative mutation of KIF1A, SPG30 gene. Sci. Rep..

[69] Nitta R., Kikkawa M., Okada Y., Hirokawa N. (2004). KIF1A Alternately Uses Two Loops to Bind Microtubules. Science.

[70] Erlich Y., Edvardson S., Hodges E., Zenvirt S., Thekkat P., Shaag A., Dor T., Hannon G.J., Elpeleg O. (2011). Exome sequencing and disease-network analysis of a single family implicate a mutation in *KIF1A* in hereditary spastic paraparesis. Genome Res..

[71] Schule R., Kremer B.P.H., Kassubek J., Auer-Grumbach M., Kostic V., Klopstock T., Klimpe S., Otto S., Boesch S., van de Warrenburg B.P. (2008). SPG10 is a rare cause of spastic paraplegia in European families. J. Neurol. Neurosurg. Psychiatry.

[72] Yonekawa Y., Harada A., Okada Y., Funakoshi T., Kanai Y., Takei Y., Terada S., Noda T., Hirokawa N. (1998). Defect in Synaptic Vesicle Precursor Transport and Neuronal Cell Death in KIF1A Motor Protein–deficient Mice. J. Cell Biol..

[73] Adzhubei I.A., Schmidt S., Peshkin L., Ramensky V.E., Gerasimova A., Bork P., Kondrashov A.S., Sunyaev S.R. (2010). A method and server for predicting damaging missense mutations. Nat. Methods.

[74] Altschul S.F., Gish W., Miller W., Myers E.W., Lipman D.J. (1990). Basic local alignment search tool. J. Mol. Biol..

[75] Kikkawa M., Sablin E.P., Okada Y., Yajima H., Fletterick R.J., Hirokawa N. (2001). Switch-based mechanism of kinesin motors. Nature.

[76] Nescav Syndrome Disease: Malacards—Research Articles, Drugs, Genes, Clinical Trials. https://www.malacards.org/card/nescav_syndrome.

[77] Okamoto N., Miya F., Tsunoda T., Yanagihara K., Kato M., Saitoh S., Yamasaki M., Kanemura Y., Kosaki K. (2014). KIF1A mutation in a patient with progressive neurodegeneration. J. Hum. Genet..

[78] Chitty L.S., Robb S., Berry C., Silver D., Baraitser M. (1996). PEHO or PEHO-like syndrome?. Clin. Dysmorphol..

[79] Somer M. (1993). Diagnostic criteria and genetics of the PEHO syndrome. J. Med. Genet..

[80] Yiş U., Hız S., Anal O., Dirik E. (2011). Progressive encephalopathy with edema, hypsarrhythmia, and optic atrophy and PEHO-like syndrome: Report of two cases. J. Pediatr. Neurosci..

[81] Richards S., Aziz N., Bale S., Bick D., Das S., Gastier-Foster J., Grody W.W., Hegde M., Lyon E., Spector E. (2015). Standards and guidelines for the interpretation of sequence variants: A joint consensus recommendation of the American College of Medical Genetics and Genomics and the Association for Molecular Pathology. Anesthesia Analg..

[82] Levy S.E., Mandell D.S., Schultz R.T. (2009). Autism. Lancet.

[83] Tomaselli P.J., Rossor A.M., Horga A., Laura M., Blake J.C., Houlden H., Reilly M.M. (2017). A *de novo* dominant mutation in *KIF1A* associated with axonal neuropathy, spasticity and autism spectrum disorder. J. Peripher. Nerv. Syst..

[84] Kurihara M., Ishiura H., Bannai T., Mitsui J., Yoshimura J., Morishita S., Hayashi T., Shimizu J., Toda T., Tsuji S. (2020). A Novel de novo KIF1A Mutation in a Patient with Autism, Hyperactivity, Epilepsy, Sensory Disturbance, and Spastic Paraplegia. Intern. Med..

[85] Huang Y., Jiao J., Zhang M., Situ M., Yuan D., Lyu P., Li S., Wang Z., Yang Y., Huang Y. (2021). A study on KIF1A gene missense variant analysis and its protein expression and structure profiles of an autism spectrum disorder family trio. Zhonghua Yi Xue Yi Chuan Xue Za Zhi.

[86] Demily C., Lesca G., Poisson A., Till M., Barcia G., Chatron N., Sanlaville D., Munnich A. (2018). Additive Effect of Variably Penetrant 22q11.2 Duplication and Pathogenic Mutations in Autism Spectrum Disorder: To Which Extent Does the Tree Hide the Forest?. J. Autism Dev. Disord..

[87] Schimert K.I., Budaitis B.G., Reinemann D.N., Lang M.J., Verhey K.J. (2019). Intracellular cargo transport by single-headed kinesin motors. Proc. Natl. Acad. Sci. USA.

[88] Derr N.D., Goodman B.S., Jungmann R., Leschziner A.E., Shih W.M., Reck-Peterson S.L. (2012). Tug-of-War in Motor Protein Ensembles Revealed with a Programmable DNA Origami Scaffold. Science.

[89] Hariadi R.F., Sommese R.F., Sivaramakrishnan S. (2015). Tuning myosin-driven sorting on cellular actin networks. Elife.

[90] Toropova K., Mladenov M., Roberts A.J. (2017). Intraflagellar transport dynein is autoinhibited by trapping of its mechanical and track-binding elements. Nat. Struct. Mol. Biol..

[91] Driller-Colangelo A.R., Chau K.W., Morgan J.M., Derr N.D. (2016). Cargo rigidity affects the sensitivity of dynein ensembles to individual motor pausing. Cytoskeleton.

[92] Furuta K., Furuta A., Toyoshima Y.Y., Amino M., Oiwa K., Kojima H. (2012). Measuring collective transport by defined numbers of processive and nonprocessive kinesin motors. Proc. Natl. Acad. Sci. USA.

[93] Cong D., Ren J., Zhou Y., Wang S., Liang J., Ding M., Feng W. (2021). Motor domain-mediated autoinhibition dictates axonal transport by the kinesin UNC-104/KIF1A. PLOS Genet..

[94] Ren J., Wang S., Chen H., Wang W., Huo L., Feng W. (2018). Coiled-coil 1-mediated fastening of the neck and motor domains for kinesin-3 autoinhibition. Proc. Natl. Acad. Sci. USA.

[95] Kanai Y., Wang D., Hirokawa N. (2014). KIF13B enhances the endocytosis of LRP1 by recruiting LRP1 to caveolae. J. Cell Biol..

[96] Hanada T., Lin L., Tibaldi E.V., Reinherz E.L., Chishti A.H. (2000). GAKIN, a Novel Kinesin-like Protein Associates with the Human Homologue of the Drosophila Discs Large Tumor Suppressor in T Lymphocytes. J. Biol. Chem..

[97] Miki H., Setou M., Kaneshiro K., Hirokawa N. (2001). All kinesin superfamily protein, KIF, genes in mouse and human. Proc. Natl. Acad. Sci. USA.

[98] Frontiers|Breaking Boundaries in the Brain—Advances in Editing Tools for Neurogenetic Disorders. https://www.frontiersin.org/articles/10.3389/fgeed.2021.623519/full.

[99] Westhaus A., Cabanes-Creus M., Rybicki A., Baltazar G., Navarro R.G., Zhu E., Drouyer M., Knight M., Albu R.F., Ng B.H. (2020). High-Throughput *In Vitro*, *Ex Vivo,* and *In Vivo* Screen of Adeno-Associated Virus Vectors Based on Physical and Functional Transduction. Hum. Gene Ther..

[100] Kim Y.G., Chandrasegaran S. (1994). Chimeric restriction endonuclease. Proc. Natl. Acad. Sci. USA.

[101] Carlson D.F., Tan W., Lillico S.G., Stverakova D., Proudfoot C., Christian M., Voytas D.F., Long C.R., Whitelaw C.B.A., Fahrenkrug S.C. (2012). Efficient TALEN-mediated gene knockout in livestock. Proc. Natl. Acad. Sci. USA.

[102] Iyama T., Wilson D.M. (2013). DNA repair mechanisms in dividing and non-dividing cells. DNA Repair.

[103] Suzuki K., Tsunekawa Y., Hernandez-Benitez R., Wu J., Zhu J., Kim E.J., Hatanaka F., Yamamoto M., Araoka T., Li Z. (2016). In vivo genome editing via CRISPR/Cas9 mediated homology-independent targeted integration. Nature.

[104] Suzuki K., Yamamoto M., Hernandez-Benitez R., Li Z., Wei C., Soligalla R.D., Aizawa E., Hatanaka F., Kurita M., Reddy P. (2019). Precise in vivo genome editing via single homology arm donor mediated intron-targeting gene integration for genetic disease correction. Cell Res..

[105] Anzalone A.V., Randolph P.B., Davis J.R., Sousa A.A., Koblan L.W., Levy J.M., Chen P.J., Wilson C., Newby G.A., Raguram A. (2019). Search-and-replace genome editing without double-strand breaks or donor DNA. Nature.

[106] Homology-Mediated End Joining-Based Targeted Integration Using CRISPR/Cas9|Cell Research. https://www.nature.com/articles/cr201776.

[107] Nishiyama J., Mikuni T., Yasuda R. (2017). Virus-Mediated Genome Editing via Homology-Directed Repair in Mitotic and Postmitotic Cells in Mammalian Brain. Neuron.

[108] Microhomology-Mediated End-Joining-Dependent Integration of Donor DNA in Cells and Animals Using TALENs and CRISPR/Cas9|Nature Communications. https://www.nature.com/articles/ncomms6560.

[109] Płoski R. (2016). Next Generation Sequencing—General Information about the Technology, Possibilities, and Limitations. Clinical Applications for Next-Generation Sequencing.

[110] Cheng E.Y., Gleason C.A., Juul S.E. (2018). 18—Prenatal Diagnosis. Avery’s Diseases of the Newborn.

